# Atheroprotective natural anti-phosphorylcholine antibodies of IgM subclass are decreased in Swedish controls as compared to non-westernized individuals from New Guinea

**DOI:** 10.1186/1743-7075-4-7

**Published:** 2007-03-20

**Authors:** Johan Frostegård, WenJing Tao, Anastasia Georgiades, Lennart Råstam, Ulf Lindblad, Staffan Lindeberg

**Affiliations:** 1 Department of Medicine, Karolinska University Hospital, Huddinge, Stockholm, Sweden; 2 Department of Psychiatry and Behavioral Sciences, Duke University Medical Center, USA; 3 Department of Clinical Sciences, Lund University, Sweden

## Abstract

**Objective:**

To determine the importance of IgM antibodies against phosphorylcholine (aPC), a novel protective factor for cardiovascular disease (CVD), in a population with a non-western life style as compared with a Swedish control group.

**Methods and results:**

Risk factors for cardiovascular disease were determined in a group of 108 individuals aged 40–86 years from New Guinea and 108 age-and sex-matched individuals from a population based study in Sweden. Antibodies were tested by ELISA. aPC IgM levels were significantly higher among New Guineans than among Swedish controls (p < 0.0001). This difference remained significant among both men and women when controlled for LDL and blood pressure which were lower and smoking which was more prevalent in New Guineans as compared to Swedish controls (p < 0.0001). aPC IgM was significantly and negatively associated with age and systolic blood pressure among Swedish controls and with waist circumference among New Guineans. aPC IgM levels were significantly higher among women than men in both groups. The proportion of the saturated fatty acid (FA) myristic acid in serum cholesterol esters was negatively but polyunsaturated eicosapentaenoic acid and also lipoprotein (a) were positively associated with aPC IgM levels.

**Conclusion:**

IgM-antibodies against PC, which have atheroprotective properties, are higher in a population from Kitava, New Guinea with a traditional lifestyle, than in Swedish Controls, and higher among women than men in both populations tested. Such antibodies could contribute to the low incidence of cardiovascular disease reported from Kitava and could also provide an explanation as to why women have a later onset of CVD than men.

## Background

The population from Kitava, Trobriand Islands, Papua, New Guinea, is virtually uninfluenced by the dietary habits and sedentary lifestyle that are common in developed countries. Even though controlled studies of atherosclerosis have not been performed in this population, available evidence indicates that cardiovascular disease is very uncommon[1]. Previous studies have indicated that this difference may be related to clear differences in measurements related to the metabolic syndrome, for example, BMI, waist circumference, blood pressure, LDL, insulin- and leptin levels are low among individuals from Kitava[2,3]. These factors could provide important clues as to why CVD is largely absent in Kitava. There are also interesting dietary differences as compared to a Western population. In Kitava, staple foods include roots, fruit, fish, coconut and vegetables. Cereals, dairy products, separated fats and refined carbohydrates, which are major staples in western countries, are absent.

Recent evidence indicates that atherosclerosis, the main cause of cardiovascular disease (CVD) is an inflammatory disease characterized by immune activation in the artery wall[4,5]. It is therefore of interest to investigate differences in atherosclerosis-related immune reactivity between individuals from Kitava and Sweden.

One such factor that could promote inflammation and immune activation in the artery wall is oxidized low density lipoprotein (OxLDL). Early studies indicated that OxLDL can induce activation of monocytes/macrophages[6], endothelial cells[6,7] and T cells[8]. OxLDL promotes inflammation also in immune competent cells from atherosclerotic lesions[9].

The role of antibodies against OxLDL (aOxLDL) has been debated[10,11]. It was originally reported that such antibodies where associated with increased risk of atherosclerosis progression and were risk factors for CVD[12,13]. In contrast, we demonstrated that aOxLDL were decreased in patients with early signs of CVD-risk as in borderline hypertension and suggested that aOxLDL was instead a protection factor[14], a notion supported by other recent reports[15,16]. The cause of these discrepancies in reports about the role of aOxLDL is not clear, but one possibility is that oxLDL is such a complicated compound that aOxLDL measurements are difficult to standardize.

Many biological effects of OxLDL are caused by platelet activating factor (PAF)-like lipids and/or lysophosphatidylcholine (LPC) in OxLDL [17-19] where a small epitope, phosphorylcholine (PC) is a major component and essential for interaction with the PAF-receptor and one important scavenger receptor that takes up oxLDL in macrophages, CD34. PC is also an immunogenic component of many bacteria including S Pneumoniae[20].

We recently demonstrated that aPC of IgM subclass is a protection factor for human atherosclerosis in patients with hypertension[21]. We here report that IgM aPC are on average much higher among individuals from Kitava than among Swedish controls, and higher in women than in men in both populations. The implications of these findings are discussed.

## Methods

### Subjects

Serum samples were obtained from 108 subjects aged 40–86 years from Kitava, Trobriand Islands, Papua New Guinea, and from 108 age-matched Swedish population based control subjects. Both surveys were approved by the Ethical committee at the University of Lund. The Kitava study was given ethical approval by the National Medical Research Advisory Committee of Papua New Guinea (PNG). It was approved by other PNG national and provincial bodies and at the community level by the inhabitants and their chiefs.

The Kitavans are primitive horticulturalists who were essentially uninfluenced by western dietary habits at the time of the survey in 1990[22]. From a total population of 2300 Kitavans, all subjects older than 50 years (n = 206) and 20% of those aged 40 ± 49 (n = 41) were eligible for this study. Informed consent was obtained through personal contact. Ages were calculated from well known historical events and were considered accurate to within 3 years for most subjects. The acceptance rate for serum sampling was only 42% and, therefore, self-selected subjects below 50 years were included. These consisted of persons excluded by the random generator but willing to take part. Individual subjects did not receive payment for their participation.

The Swedish controls consisted of Caucasians (and very few immigrants), half of whom were living in the countryside and the other half in the small town of Skara. The survey was conducted in 1993–94 among a random sample from the population census register stratified by age and sex[23]. Selected residents in Skara were invited to a health control at the Skara Health Care centre in 1993–94 (baseline examination). From each 10-year age-category between 40 and 79 years of age 150 male and 150 female residents, and in the ages 80 years and above 100 male and 100 female subjects, were selected. Of these 1400 invited subjects, 1109 (80%) participated and completed a physical examination. For the present study, one subject, matched for age and sex, was randomly chosen for each of the 108 Kitavan subjects. In both populations blood samples were taken before 9.00 a.m. after overnight fasting. Blood was centrifuged and serum was frozen in liquid nitrogen within 60 min. Serum samples were stored in -70°C until analysis.

### Clinical, lipid- and urate measurements

Standard methods were used for measurements of weight, standing height, waist circumference, triceps skinfold (TSF) thickness, upper mid-arm circumference (MAC) and serum levels of total cholesterol (TC), high density lipoprotein cholesterol (HDL), triglycerides (TG) and uric acid [23-25]. Blood pressure was measured with subjects in the sitting position. In Kitava, the measurement arm was kept parallel to the sternum, thus identical to another Swedish control population [23-25]. In contrast, in the Skaraborg survey, the measurement arm was at heart level. From other studies it is known that blood pressure is lower in the latter position[26]. Arm muscle circumference (AMC) was derived using the equation AMC = MAC - 0.1(π × TSF). TC was measured using a cholesterol-esterase/cholesterol-oxidase method with Seronorm Batch no. 50 (Nyegaard, Oslo, Norway) as a calibrator. HDL was determined after precipitation of very low density lipoprotein cholesterol and low density lipoprotein cholesterol (LDL) with dextran sulphate and magnesium chloride. TG was analysed using an enzymatic-colorimetric method. Low density lipoprotein cholesterol (LDL) was estimated according to Friedewald. Serum uric acid was determined by the uricase-peroxidase method. The fatty acid composition of serum cholesterol esters was determined by using gas liquid chromatographic methods[27]. Lipoprotein(a) (Lp(a)) was measured by the Pharmacia Apo(a) RIA method as described earlier[24].

Clinical characteristics of the two study populations are presented in Table 1.

**Table 1:** Basic characteristics of the study groups. Results are presented as means (SD) or percentages and mmol/L for lipids.

	**New Guineans (N = 108)**	**Swedish controls (N = 109)**	**P**
**aPC**	981 (283)	740 (295)	0.0001
**Age (years)**	59.9 (11.1)	59.7 (10.6)	0.85
**Sex (% males)**	70	70	0.97
**SBP (mmHg)**	118.6 (18.0)	135.3 (18.2)	0.0001
**DBP (mmHg)**	70.0 (7.1)	77.3 (9.4)	0.0001
**BMI**	18.8 (2.2)	25.8 (4.1)	0.0001
**Waist**	70.9(4.4)	87.2(10.9)	0.0001
**Smokers (%)**	75.2	24.7	0.0001
**Total Cholesterol**	5.1 (1.2)	5.8 (0.9)	0.0001
**HDL**	1.07 (0.15)	1.14 (0.26)	0.04
**LDL**	3.5 (1.1)	4.0 (0.88)	0.0001
**Triglycerides**	1.2 (0.46)	1.3 (0.77)	0.10

SBP, systolic blood pressure

DBP, diastolic blood pressure

BMI, body mass index

HDL, high density lipoprotein

LDL, low density lipoprotein

### Reagents

Polysorp F96 microtiter immuno-plates were purchased from Nunc (Roskilde Denmark), PC-BSA (Phosphorylcholine-Bovine Serum Albumin) was purchased from Biosearch Technologies, Inc (Ca, USA).

### Determination of IgM antibodies against PC

IgM antibodies to PC-BSA were determined by enzyme-linked immunosorbent assay (ELISA) essentially as described[21]. Briefly, pooled serum from medium to high-titer individuals was used as an internal standard and tested on every plate. The plateau of antibody binding was reached with the antigen concentration of 10 μg/ml. F96 microtiter polysorp plate was therefore coated with PC-BSA(10 μg/ml) 50 μl/well in PBS. Coated plates were incubated overnight at 4°C. After five washings with PBS, the plates were blocked with 2% BSA-PBS for 2 h at room temperature and washed as described above. Serum samples were diluted (1:30) in 0.2% BSA-PBS and added at 50 μl/well.

Plates were incubated overnight at 4°C and washed as described above. Alkaline phosphatase conjugated goat anti-human IgM (diluted 1:7000 in the sample buffer) were added at 100 μl/well and incubated at 4°C overnight. After five washings, color was developed by adding the alkaline phosphatase substrate (PNPP) at 100 μl/well and incubating the plates for 60 min at room temperature in the dark. The plates were read in an ELISA Multiscan Plus spectrophotometer (Molecular Devices Emax, San Francisco) at 405 nm. All samples were measured in duplicates a single assay and the coefficient of variation was below 10–15%.

### Statistical analysis

Variables were tested for normality. Groups with skewed variables were compared using the non-parametric Mann-Whitney's *U *test and categorical variables were compared using *chi-square *tests. General linear model (GLM) was applied using the statistical software SPSS version 12.02 for Windows to test analysis of variance and investigate group differences and interaction effects of group and gender on aPC IgM levels. Associations between aPC IgM levels and traditional risk factors were determined using Pearson correlations for normally distributed variables and Spearman rank correlations for skewed variables. In addition, linear regression analysis was conducted with aPC IgM levels as dependent variable. Additional analysis of co-variance were made for aPC IgM levels while adjusting for the traditional cardiovascular risk factors smoking, blood pressure (BP), body mass index (BMI), and lipid levels. Data are presented as means (standard deviation). The level of statistical significance was set at a value of *P *< 0.05.

## Results

**Figure 1:**
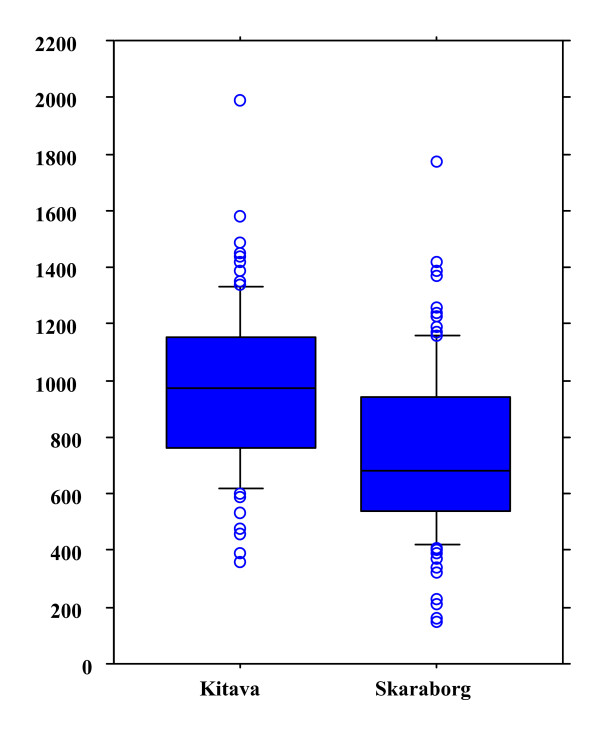
Antibody levels against phosphorylcholine (aPC) of IgM subclass, in 108 individuals from New Guinea, Kitava as compared to 109 age and sex matched controls from Skaraborg, Sweden. Antibody levels were determined as described in the Methods section.

Table 1 presents basic characteristics by group. There were striking differences between the Kitava population and the Swedish controls in aPC IgM levels as indicated in Table 1 and Figure 1. In addition to the significant difference in aPC IgM levels between the two populations, metabolic factors and blood lipids differed significantly with a more atherogenic profile in the Swedish population. In contrast, smoking was much more common among New Guineans (see Table 1).

Correlation analysis between aPC IgM levels and traditional riskfactors for all participants and for Kitava and Skaraborg populations separately are presented in Table 2. Among all studied participants, aPC IgM levels were significantly associated with systolic BP levels (r = .27, p < .001), diastolic BP levels (r = .15, p = .04), and BMI (r = .-.34, p < .001) and gender (r = .20, p < .01), whereas aPC IgM levels were not significantly related to total cholesterol, LDL or triglyceride levels. When separating the groups, significant associations were only found between aPC IgM and waist in the Kitava population (r = -.21, p = .03), and between aPC IgM and age (r = -.22, p = .02) and SBP (r = -.23, p = .01) in the Swedish population.

**Table 2:** Pearson correlation analysis of aPC IgM in association with traditional riskfactors for all study participants (n = 216) and for the Kitava (n = 108) and Skaraborg (n = 108) populations separately.

	**aPC IgM All**	**aPC IgM Kitava**	**aPC IgM Skaraborg**
**Age (years)**	-.08	.03	-.22*
**SBP (mmHg)**	-.27***	.001	-.23**
**DBP (mmHg)**	-.15*	-.005	-.03
**BMI**	-.34***	-.16	-.05
**Smoking (no/yes)**	.23**	.10	-.03
**Waist**	-.36***	-.21*	-.12
**Total Cholesterol**	-.11	.06	-.06
**HDL**	-.07	-.18	.10
**LDL**	-.11	.09	-.10
**Triglycerides**	-.03	.14	.01

* p < .05, ** p < .01, ***p < .001

SBP, systolic blood pressure

DBP, diastolic blood pressure

BMI, body mass index

HDL, high density lipoprotein

LDL, low density lipoprotein

A multivariate linear regression including group (Kitava, Sweden), gender (female, male), presently smoking (no/yes), systolic BP, BMI and LDL lipid levels and triglycerides was performed and results are presented in Table 3. Group, gender and systolic BP were independent predictors of aPC IgM levels and together they explained 22% of the variance of aPC IgM levels.

**Table 3:** Multiple regression analysis with aPC IgM as dependent variable and groups (populations) as well as traditional cardiovascular risk factors as independent variables

	**Beta**	**SE of Beta**	**P**
**Group (New Guinea/Sweden)**	.16	.07	.02
**Gender (male/female)**	.15	.05	.002
**Smoking (no/yes)**	.01	.05	.78
**Age (years)**	.01	.007	.84
**SBP (mmHg)**	-.004	.001	.01
**DBP (mmHg)**	.005	.003	.07
**BMI**	-.01	.007	.09
**LDL**	-.01	.02	.70
**Triglycerides**	.03	.03	.37

SBP, systolic blood pressure

DBP, diastolic blood pressure

BMI, body mass index

HDL, high density lipoprotein

LDL, low density lipoprotein

Table 4 presents the results from interaction analysis of variance (group by gender). Main effects as well as group by gender interactions are presented for all variables. As shown above in Table 1, there was a group main effect on aPC IgM levels (p < .001), with significantly higher levels of aPC IgM in both the New Guinea men and women as compared to the Swedish age and gender matched population. There was also a significant effect of gender on aPC IgM levels (p < .0001), with women having higher aPC IgM levels as compared to men. There was no significant interaction between gender and group on aPC IgM levels (p = .82). There was a significant group effect on systolic BP (p < .0001), reflecting that New Guineans overall had lower systolic BP as compared to the Swedish population. There was no significant main effect of gender on systolic BP or interaction between gender and group. Diastolic BP only showed a trendwise difference for the group comparison (p = .07), whereas the gender main effect and interaction between gender and group was significant, with Swedish men having higher diastolic BP as compared to all other groups (see Table 3). There was a significant main effect of group on BMI, with the New Guineans having significantly lower BMI as compared to the Swedish population (p < .0001). There was also a main effect of gender, with women having lower BMI as compared to the men, whereas the gender × group interaction on BMI levels did not reach significance. Waist was significantly lower in the Kitava population (p < .001), and women had lower waist measure as compared to men (p < .001). There was a significant main effect of group on both total cholesterol and LDL levels, with the New Guineans having significant lower total cholesterol and LDL levels as compared the Swedish population. In addition, there was a main effect of gender as well as a significant interaction between gender and group, with the New Guinean men having significant lower total cholesterol and LDL levels as compared to the other groups. There was no group effect on HDL levels, but there was a significant gender effect, with women having higher HDL levels as compared to men. In addition, there was a significant group effect on LDL levels, as well as a main gender effect and a gender by group interaction on LDL levels, with New Guineans having lower LDL than the Swedish controls, and overall women having lower LDL than men and finally New Guinean men having significant lower levels as compared to all other groups. There was no significant difference between the groups in triglycerides, nor did the sexes differ in triglyceride levels. In addition, smoking was significantly more common among New Guineans as compared to the Swedish controls, but there was no main effect on gender (equal amount of men and women smoked). However, there was a difference in smoking pattern between the populations, with women from Sweden smoking the least (12%) compared to all other groups, and women from New Guinea having the highest frequency of smokers (88%).

**Table 4:** Results are presented as means (SD) or percentages and mmol/L for lipids.

	**New Guinean**		**Swedish**		***P***		
	**Women (N = 32)**	**Men (N = 76)**	**Women (N = 32)**	**Men (N = 77)**	**Group**	**Gender**	**Group × Gender**
**aPC IgM**	1072 (309)	943(265)	842 (324)	698 (273)	.001	.0001	.82
**SBP (mmHg)**	123.1 (18.8)	116.7 (17.3)	135.6 (18.5)	135.2 (18.2)	.0001	.22	.28
**DBP (mmHg)**	70.2 (8.1)	69.9 (6.7)	73.8 (8.8)	78.8 (9.3)	.07	.0001	.04
**BMI**	17.4 (2.0)	19.4 (2.0)	25.8 (5.3)	25.9 (3.4)	.0001	.05	.08
**Waist**	67.7(4.8)	74.1(4.1)	80.7(12.8)	93.8(9.3)	.0001	.0001	.007
**Total Cholesterol**	5.9 (1.3)	4.7 (0.94)	5.8 (0.93)	5.8 (0.96)	.004	.0001	.0001
**HDL**	1.20 (0.26)	1.0 (0.23)	1.24 (0.31)	1.1 (0.23)	.08	.001	.50
**LDL**	4.16 (1.16)	3.2 (0.90)	4.0 (0.84)	4.1 (0.90)	.004	.0001	.0001
**Triglycerides**	1.30 (0.52)	1.16 (0.44)	1.23 (0.53)	1.38 (0.79)	.38	.92	.10
**Smoking (%)**	88	70	12	30	.001	.98	.008

SBP, systolic blood pressure

DBP, diastolic blood pressure

BMI, body mass index

HDL, high density lipoprotein

LDL, low density lipoprotein

Analysis of covariance (ANCOVA) was performed to investigate if the difference in aPC IgM between the New Guinean and the Swedish group would remain significant after controlling for the CVD risk factors that were significantly different between the groups. The difference in aPC Igm between the New Guinean and Swedish population remained significant after adjusting for smoking, systolic BP, total cholesterol and BMI (F(1, 202) = 5.7, p = .02).

In addition to the 108 individuals from Kitava that were matched with Swedish controls over 40, we also had access to an additional 68 Kitavan individuals under the age of 40. In the whole group from Kitava (comprising of 176 individuals), additional separate analysis and measurements were made, that were not accessible for the Swedish control population. aPC IgM levels were associated with waist (r = -.24, p = .002) and upper arm muscularity as represented by AMC (r = -.17, p = .03) and MAC (r = -.16, p = .04). However, BMI was not significantly associated with aPC in this population (r = -.10, p = .20). Among blood lipids, only apo (a) was associated with aPC IgM (r = .23, p = .004). BP measurements, electrolytes, and transaminases, were not associated with aPC. There were significant associations between aPC and certain fatty acids (FA) in serum cholesterol esters: saturated FA C 14:0 (myristic acid) was negatively (r = -.17, p = .03) but polyunsaturated FA C 20:3n-6 was positively associated with aPC levels (r = .21, p = .007). C14:0 and C20:3n-6 were significantly (and negatively) associated with each other (r = -.45, p = 001). Kitavans over the age of 40 had lower levels of aPC than those under 40 years old (.98(.28) vs 1.07(.23), p = .03) but this association did not quite reach significance when aPC IgM and age as a continuous variable was used (r = -.14 p = 0.06). A stepwise regression model with aPC IgM as dependent variable, showed Lp(a), waist, and CE 20:3 to be significantly and independently associated to aPC IgM.

## Discussion

The existence of antibodies against PC in humans has been known for decades[20], though their clinical role and function are not well characterized. We recently reported that aPC IgM were negatively associated with atherosclerosis development in hypertensives. Furthermore, a high level of aPC of IgM subclass (both at the 75^th ^and 90^th ^percentile) is associated with a favourable outcome in ultrasound measurements of atherosclerosis prospectively and thus is a protection factor for atherosclerosis development in both women and men with hypertension[21].

We here report that levels of IgM autoantibodies to PC (aPC) are significantly higher in a population from Kitava, New Guinea with a traditional lifestyle, as compared to Swedish controls, matched for age and sex. CVD is virtually unknown among individuals from Kitava[22], and we therefore hypothesize that atheroprotective aPC of IgM subclass may contribute to this.

Oxidized or other forms of modified Low Density Lipoprotein (OxLDL) are factors that are widely believed to be of importance in atherogenesis since they promote immune activtion, inflammation and also apoptosis and necrosis[10]. We demonstrated in early studies that OxLDL can activate monocytes/macrophages and endothelial cells[6] and T cells[8,28]. Further studies have lead to clarification of one underlying mechanism by which oxLDL promotes immune activation, namely by platelet-activating factor (PAF) and PAF-like lipids in oxLDL which all share PC as a major epitope binding to the PAF-receptor [17,18,29]. OxLDL is taken up in macrophages through specific scavenger receptors, and one major such receptor, CD 36, has PC as its ligand[30]. Furthermore, PC is known to be immunogenic, present on important human pathogens like S Pneumoniae[20], and also apoptotic cells expose this antigen that is normally cryptic[31]. PC is therefore a highly relevant compound in atherosclerosis.

In mice, clonally expanded T15 antibodies recognizing PC which confer protection against lethal infection with S. Pneumoniae were described already in the 1980s[32]. Such T cell independent antibodies are produced by a B cell subset, B1 cells, as a part of the innate immune system[33].

In mice models of atherosclerosis, aOxLDL and aPC appear to be raised, and thus these antibodies, could be described as risk markers, associated with atherosclerosis in this context[34]. Still, several investigators have demonstrated that immunization with oxLDL [34-36] or S Pneumoneae[37] attenuates atherosclerosis development in such models in parallel with further raised aOxLDL and aPC levels respectively – albeit from an already high level of such antibodies[34] and passive transfer of aPC ameliorates atherosclerosis development in a mouse model][38].

The cause of these differences between mouse atherosclerosis models and human atherosclerosis is presently unclear but could indicate that the mouse models for atherosclerosis only reflect a subfraction of human atherosclerosis. Even though mouse models of atherosclerosis have increased our knowledge about the role of the immune system in atherosclerosis, they do not develop the same type of CVD with atherothrombosis and plaque rupture as humans[39].

In humans the role of aPC in general and particularly infectious diseases is unclear, and in one study, aPC was even reported to be associated prospectively with an increased risk of death in pneumonia caused by this infectious agent[40].

Since CVD is virtually unknown in Kitava, our findings suggest that high levels of aPC IgM could function as a protective factor in these individuals, contributing to protection against atherosclerosis and CVD. This notion is also supported by the fact that the high levels of aPC IgM among New Guineans was independent of other risk factors that are more prevalent in Swedish controls including blood pressure, lipids and BMI-related measurements. Our preliminary experiments indicate that polyclonal human aPC of IgM subclass can inhibit uptake of oxLDL in macrophages, suggesting an underlying mechanism by which aPC IgM could be atheroprotective (unpublished observation). Other possibilities include inhibition of proinflammatory effects medicated by PAF-like lipids generated during LDL-oxidation.

We confirm and extend a previous finding in hypertensives, where men had lower aPC IgM levels than women[21]. Both among New Guineans and Swedish controls, men had significantly lower levels of aPC IgM as compared to women. We thus hypothesize that these findings could be one underlying factor that contributes to the lower risk of CVD in women as compared to men of similar age.

PC is an intriguing molecule from an immunological point of view, with several properties that could in principle both promote and protect against disease depending on pathogen and type of inflammatory reaction. For example, PC-exposing compounds could elicit at low grade chronic inflammation and in principle thus also contribute to atherosclerosis, while protecting against more acute infections and inflammation[20,41]. In line with this, PC-containing filarial nematodes, can achieve longevity in the infected host by suppressing and modulating the host immune response[42].

The underlying mechanisms that cause the decreased aPC IgM levels in Sweden (or raised levels in New Guinea, depending on perspective), could have different non-mutually exclusive explanations including both life style factors and underlying genetic variations. However, genetics are less likely to explain the findings, considering the emergence of cardiovascular disease and diabetes in neighbouring populations after westernization [43-45]

It should also be noted that the cause of the low antibody levels against these antigens in individuals with increased atherosclerosis could be caused by consumption into the atherosclerotic lesions which are known to contain OxLDL epitopes, or formation of immune complexes containing PC or OxLDL.

Among life style factors, differences in diet and exposure to infections are interesting possibilities.

There were some potentially interesting associations between dietary fat [as measured by serum cholesterol ester fatty acids (CE-FA)] and aPC IgM levels. The diet in Kitava is rich in saturated myristic acid which is reported to raise LDL-levels and which, in our study, was negatively associated with aPC levels. In contrast a polyunsaturated FA, dihomo-gama-linolic acid 20:3n-6 was positively associated with aPC. It could be hypothesized that exposure to easily oxidized FA e g in the gut immune system could elicit more robust aPC IgM levels, in contrast to saturated FA which are not oxidized. However, a caveat is that only some and not all FA showed this association pattern and further studies are needed to clarify if these associations are of importance. It may be relevant in this context to consider the fact that serum lipid levels are not optimal in the Kitavans, and higher than in some other non-western populations[24]. which partly could be due to the relatively high intake of saturated FA from coconut. The apoplipoprotein B to A1 ratio was actually higher in Kitava than in a Swedish control population analysed at the same laboratory[24].

There was a strong positive association between aPC IgM levels and Lp(a), which is interesting, since this lipoprotein is known to react with a monoclonal antibody against oxLDL that also recognized PC, and much PC exposed on LDL in the circulation is present on Lp(a). Mean levels of Lp(a) did not differ between Kitava and Sweden[24]. Since this lipoprotein has potentially atherogenic properties, it is possible that raised levels of Lp(a) promote a robust immune reaction at least in individuals from Kitava. Whether such associations are present also in a Swedish population is not known.

Pathogens exposing immunogenic PC include both bacteria like S Pneumoniae and various forms of nematodes, and one possibility that deserves further exploration is that increased exposure to some infections in New Guinea promoting aPC-related immune response could protect against atherosclerosis.

Smoking was significantly more prevalent among new Guineans of both sexes, than in Swedish controls. Cigarette smoking is associated with an increased risk of stroke and IHD within most westernized populations, but in countries like Japan and China the prevalence of CVD has been low, despite extensive smoking. It is therefore possible that smoking is better tolerated in individuals who are not exposed to other risk factors.

In summary, our findings indicate that high titers of IgM antibodies against PC, which we recently described as being a protective factor for atherosclerosis[21], are more prevalent among individuals from Kitava, New Guinea who live a traditional life style, as compared to age and sex-matched Swedish controls. This could contribute to the low prevalence of CVD apparently present in Kitava.
